# Supplementary material for: Plant-made polio type 3 stabilized VLPs—a candidate synthetic polio vaccine
Source: Nat Commun. 2017 Aug 15;8:245. doi: 10.1038/s41467-017-00090-w (PMC5557999; doi:10.1038/s41467-017-00090-w)
Supplement: Supplementary file 1 — Supplementary Information [file 41467_2017_90_MOESM1_ESM.pdf]

File Name: Supplementary Information
Descriptions: Supplementary Figures, Supplementary Table and Supplementary Reference

File Name: Peer Review File
Descriptions:

Supplementary data

Supplementary Table 1: Cryo-EM data collection and refinement statistics.

	PV3 SktSC8 sVLP	PV3 SktSC8 sVLP + GPP3
**Data Collection**		
Microscope	FEI Titan Krios	FEI Tecnai Polara G2
Detector	FEI Falcon II	Gatan K2 Summit
Filter		Gatan GIF Quantum
Pixel size (Å)	1.10	1.35
Defocus range (µm)	-2.8 to -1.0	-2.8 to -0.8
Voltage (kV)	300	300
Electron dose (e^{-} $Å^{-2}$)	50	30
**Reconstruction**		
Number of particles	4046	2060
Software	RELION 1.3	RELION 1.3
Symmetry	I1	I1
Accuracy of rotations (°)	0.370	0.365
Accuracy of translations (pixels)	0.5	0.4
Final resolution (Å)	3.6	4.1
Map sharpening B-factor ($Å^2$)	-181.3	-126.2
**Atomic model refinement**		
Software	Phenix	Phenix
Resolution limit (Å)	3.6	4.1
Number of atoms	5892	5922
Protein atoms	5892	5892
Other atoms		30
Residues	747	747
Map CC (around atoms)	0.821	0.825
RMS bond lengths (Å)	0.01	0.01
RMS bond angles (°)	0.87	0.79
**Validation**		
Clashscore, all atoms (percentile)	3.10 (98th percentile)	3.34 (97th percentile)
Rotamer outliers (%)	0.15	0.15
C-beta deviations >0.25 Å	0	0
Ramachandran plot (%)		
Favored	93.2	91.9
Allowed	6.8	8.1
Outliers	0.0	0.0
MolProbity score (percentile)	1.54 (94th percentile)	1.61 (92nd percentile)

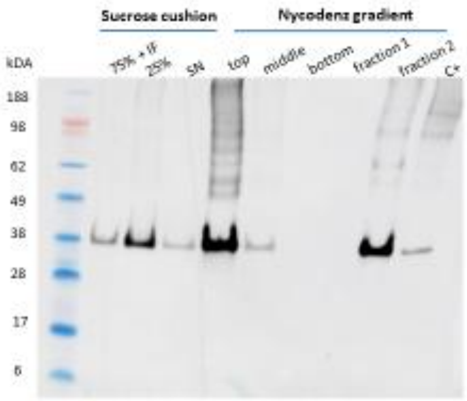

Supplementary Figure 1: Uncropped image of western blot used to create Figure 1 panels (c) and (e).

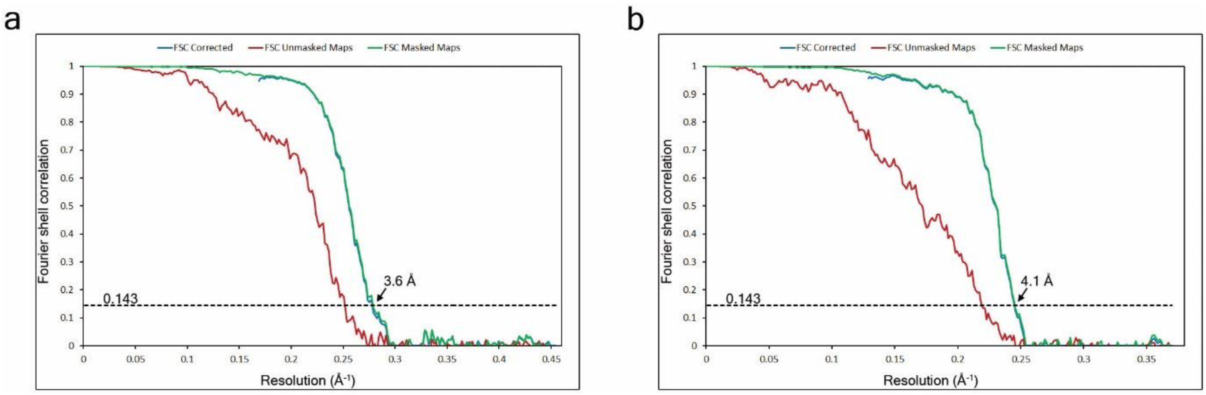

Supplementary Figure 2: Cryo-EM resolution analysis of stabilized poliovirus type 3 SC8 VLPs. FSC curves of the final 3D reconstructions for (**a**) PV3 SktSC8 and (**b**) PV3 SktSC8 + GPP3 sVLPs, obtained using gold-standard refinement using RELION, marked with the resolution corresponding to a Fourier shell correlation (FSC) of 0.143 (dashed line). Based on a FSC cut-off criteria of 0.143 the resolution of the PV3 SktSC8 and PV3 SktSC8 + GPP3 sVLPs was 3.6 Å and 4.1 Å respectively.

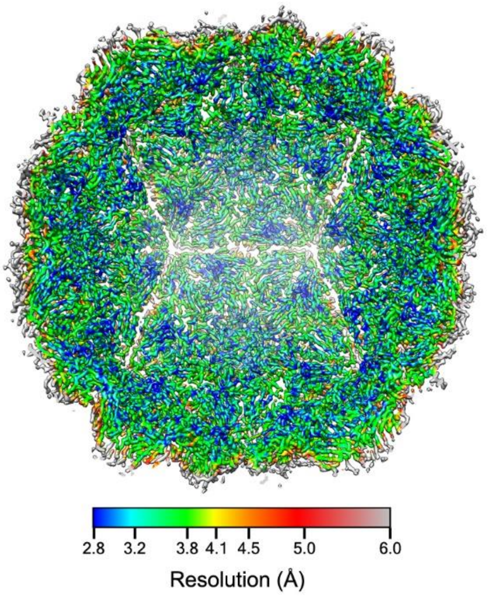

Supplementary Figure 3: Resolution analysis of PV3 SktSC8 VLP cryo-em density map. A central slice through the SktSC8 VLP (as in Figure 3c) is viewed along the 2-fold axis. The final PV3 SktSC8 VLP map was analysed by ResMap [1], and the distribution of local resolution is shown coloured from blue (2.8 Å) to grey (6.0 Å). Most of the stabilized VLP has a resolution better than 4.0 Å.

1. Kucukelbir, A., F.J. Sigworth, and H.D. Tagare, *Quantifying the local resolution of cryo-EM density maps.* Nat Methods, 2014. **11**(1): p. 63-5.
